# The cultural turn in understanding ‘addiction’

**DOI:** 10.1016/j.drugpo.2024.104473

**Published:** 2024-06-13

**Authors:** Masoomeh Maarefvand, Maziyar Ghiabi

**Affiliations:** a Substance Abuse and Dependence Research Center, University of Social Welfare and Rehabilitation Sciences, Tehran, Iran; b University of Exeter, Exeter, UK

**Keywords:** Cultural turn, Critical drug studies, Epistemology, Global south, Iran, Addiction, Habit, Useable past, Drug history, Addiction history, Decolonisation

## Abstract

In this essay we want to foreground a question: what happens to ‘addiction’ when we take seriously cultural scripts informing its trajectories? Can this bring us to unthink addiction as problematic notion and move it onto new paradigms that fit better the now acknowledged fluidity and pluralistic episteme of ‘addiction’ and more broadly of chronic life conditions? Indeed, ‘addiction’ has become a pivotal concept in the contemporary world. A powerful diagnostic framework in interpreting human behaviour, for some ‘addiction’ has become the ‘new normal’ with chronic relations with different things such as food, sex, gambling, and mind-altering substances touching upon the lifestyle of a majority of individuals, making everyone ‘addicts in practice’. Perhaps this has something to do with the constituent force that ‘habit’ – as in ‘addiction’ – has in defining our present and future. Though ‘addiction’ goes beyond the question of mind-altering drugs, the politics of ‘addiction’ is intimately tied to substances such as opioids and opiates, cocaine, cannabis, and psychedelics that have been the object of durable systemic political control and security repression. Contextually the line between licit/illicit substances is softening and blurring, the ‘dual’ purpose that drugs serve is now recognised in scientific and popular analysis moving the question of ‘addiction’ beyond the medicine/drug dichotomy. Yet, culture is generally absent in understanding ‘addiction.’ When it is referred to, this happens in diminutive terms limited to Anglo-American modern culture. Culture matters and it matters with different weights and measures as it moves across the world. There are cultural environments of health informed by practices and epistemologies of well-being that have evolved in lines opposites from or only intersecting with the Anglo-American, and generally Western, world. Exploring these spaces and cultural scripts enables our scholarship on drugs and ‘addiction’ to move the barycentre of discussion towards novel considerations around the historical trajectories and potential futures of our diagnostic terms and policy interventions.

‘You are my ball, and you run under the decree of my stick. Though I am making you to run, I am still running in your track.’

Rumi, Divan-e Shams, Ghazal no. 322

‘Addiction’ has become a pivotal concept in the contemporary world. A powerful diagnostic framework in interpreting human behaviour, for some ‘addiction’ has become the ‘new normal’ (Park, 2021) with chronic relations with different things such as food, sex, gambling, and mind-altering substances touching upon the lifestyle of a majority of individuals (Sussman, Lisha & Griffiths, 2011), making everyone ‘addicts in practice’ (Fraser, Moore & Keane, 2014). Perhaps this has something to do with the constituent force that ‘habit’ – as in ‘addiction’ – has in defining our present and future. Though ‘addiction’ goes beyond the question of mind-altering drugs, the politics of ‘addiction’ is intimately tied to substances such as opioids and opiates, cocaine, cannabis, and psychedelics that have been the object of durable systemic political control and security repression. Contextually the line between licit/illicit substances is softening and blurring, the ‘dual’ purpose that drugs serve is now recognised in scientific and popular analysis (Herzberg, 2020; Hardon & Sanabria, 2017) moving the question of ‘addiction’ beyond the medicine/drug dichotomy (Ghiabi, 2021).

Culture is generally absent in understanding ‘addiction.’ When it is referred to, this happens in diminutive terms limited to Anglo-American modern culture (Brodie & Redfield, 2002). Culture matters and it matters with different weights and measures as it moves across the world. There are cultural environments of health informed by practices and epistemologies of well-being that have evolved in lines opposites from or only intersecting with the Anglo-American, and more generally Western, world. Exploring these spaces and cultural scripts enables our scholarship on drugs and ‘addiction’ to move the barycentre of discussion towards novel considerations around the historical trajectories and potential futures of our diagnostic terms and policy interventions.

In this essay we want to foreground a question: what happens to ‘addiction’ and ‘drugs’ when we take seriously those cultural scripts informing their trajectories? Can this bring us to unthink these problematic notions (Lancaster, Duke & Ritter, 2015) and move them onto new paradigms that fit better the now acknowledged fluidity and pluralistic episteme of ‘addiction’ (Fraser, Moore & Keane, 2014) and more broadly of chronic life conditions? To dwell on such an experimental thought, we invite the readers to come along for an unusual journey to Iran, a country that has had a past and present of thought and policy experimentation on ‘addiction’ that goes back to at least the 15th century; and which in the 20th and 21st centuries have suffered public health harms (HIV and overdose epidemics, mass incarceration) linked to drugs and addiction policy. For its rich history in thinking and policymaking on addiction, Iran’s history represents an important instance of ‘usable past,’ one that is latent in the scholarships of global health or international policy regimes.

The essay uses primary research material collected in a nationwide study carried out by the authors on the subject of lived experience of ‘addiction.’ The study focused on five major urban areas of Iran carrying out life-trajectory interviews with an inclusive generational, gender, and ethno-religious in scope. The study has been supplemented by archival and conceptual inquiry on the question of ‘addiction’ by the authors’ respective research on social work and addiction epistemologies.

Initially, our intervention engages with the specific case of opium and opiates. This may seem to imply that the cultural turn of addiction to which we refer may be dependent on the ‘domesticity’ of a drug to a specific cultural environment, such as opium in Iran. Yet, a similar line of inquiry can be advanced in relation to alcohol, which though thought of as exogenous to the cultural practices of Iran (and the Islamic world), has indeed had a long and rich history, including in its health/moral counterweight of ‘alcoholism.’ We use these two categories to extend our invitation for a cultural turn.

In a treaty titled *Resaleh-ye Afyuniyeh* (The Opium Treaty), the physician and essayist ‘Emad al-Din Mahmud Bin Mas’ud Shirazi (d. 1515) introduces his chronic habit of opium use with the word ‘addiction [*e’tiyad*],’ which is currently used to refer to the modern condition in the Persian language. His-discussion of this medical condition is informed by a multi-layered analysis of individual, environmental, politico-economic, and cultural circumstances. For instance, Shirazi provided a contextual description of the chaotic violence that had occurred in the region he lived in and the way they affected people’s well-being and safety amidst the onslaught of religious repression and the fallout of the economy following a change in dynastic rule in late 15th century Iran. ‘[People] have recourse to opium in order to suppress their distress,’ he wrote in one section of the book (Shirazi 1591 [?] - 2009). In another section he refers to the widespread cultural practice of opium taking during Ramadan, the holy month of the Muslim calendar. The physician reflects upon how to manage one’s religious beliefs and practices with the chronic health condition of ‘addiction’ (Ghiabi, 2022b). The tone of his speak does not fundamentally diverge from that of advocates of safer supply and humanistic harm reduction (Tyndall, 2020).

We encountered similar considerations by people who use drugs and those who profess suffering from ‘addiction’ in our nationwide study. Our most recent study collected life trajectories among long-term drug users in five regions of Iran. What emerged from analysing the material is the rooted and legitimate place of certain substances, especially opium, in the everyday life of the communities we engaged with. For instance, in the life trajectory of a 31-year-old man living in Mashhad, Iran’s second largest city and main pilgrimage site, in the region of Khorasan which borders with Afghanistan, he told us about the way his father considered his relation to opium and other drugs: ‘My father used opium, he didn’t have that kind of addiction…according to himself, he would use it for medicine, for illness, for a cold or cough, and so on…if he was sick or if we were sick, he would puff on our faces…’ This account is reinforced by several other stories of opium being used as a medicine in continuity with long-established practices in Eastern and Central Iran. As a social practice, using drugs is part of the experience and rituals of intimacy, friendship, hospitality, mourning, well-being, and fun throughout many regions. Funeral and wedding ceremonies are sites where opium and other illicit substances are part of the ceremony, the absence of which could be interpreted as a sign of disrespect towards guests. Other specific cultural practices extend the symbolism of opium use (but, in recent decades, drug use in general) with the coming of age of adulthood and the active participation in societal life, much in contrast with notions of marginalisation and pariahdom that is associated with illicit drugs.

But what if we consider substances other than opium and opioids, for instance alcohol? Alcohol has an equally long and unconventional history, one that since the Islamisation of Iran starting from the 7–8th century CE has been associated with attempts at its prohibition. But this prohibition has never resulted in an effective eradication of alcohol culture, which flourished to its apogee in its literary celebrations of world-known poets such as Omar Khayyam (11–12th century), Hafez (13th century), and in modern times in the drinking culture that has existed legally and illegally, before and after the Islamic Revolution of 1979, which brought about its total prohibition (Matthee, 2023). For instance, alcohol drinking in contemporary Iran maintains rituals that are distinctive from drinking culture in Europe or the Americas, though cross-contamination with Western practices is also very visible. In our nation-wide research project, many participants did not consider alcohol to be a problematic substance even though alcohol drinking and alcoholic beverages are subject to a strict criminalised code, with heavy-handed punishments (i.e. incarceration, lashes, monetary fines, and in recidivous cases, the death sentence); and they are also *haram* (forbidden) in Islamic jurisprudence therefore deemed unethical and socially degenerate. When asked about their experiences with substance use, participants typically excluded alcohol from the list of substances they felt they needed to explain. Many had experienced alcohol very early in their lives, often long before trying any other substances, including mind-altering pharmaceuticals. Drinking alcohol gave them the belief that they had ‘grown up.’ A 34-year old man from Isfahan recalled,

‘I drank alcohol when I was 10 or 11 years old. It made me feel like “I’ve grown up…” The first time I drank was with an uncle who is 4 or 5 years older than me; we drank together despite the age difference. I had two shots of *araq* (distilled raisin with 40–50 % alcohol level). I felt nauseous and dizzy, but on the other hand, I also felt good, like “Hey! I drank alcohol, I can do this!” I had a strong desire to do it… I wanted to show that I had grown up. After some time, drinking alcohol became so normal that at home and at parties, nobody really bothered me about it…’

Another example is provided a 48-year-old man from Quchan a city in close proximity with the border with Turkmenistan. He described the very pleasant feeling he experienced the first time he consumed alcohol as follows,

‘The first time I drank alcohol, it was really great, I really, really loved it and didn’t want to leave that state because I had a void in my life, there were conflicts that constantly troubled my mind, troubled my thoughts, and by drinking that alcohol, I became very calm, very relaxed, very empty, it was really good – I mean, mentally, I truly reached zero! All the thoughts and worries went away, and it calmed me down a lot, which I think is why, even though I haven’t consumed anything for many years now, whenever it comes up, that pleasant memory comes back to me…’

The descriptions above are not imputable to prohibition boosting the fantasies of intoxication and its powers or to a trend towards Westernised lifestyle models, which in many urban settings have gained ground. In fact, the rituality of drinking, with its performative nature made of poetic and expressive toasts and pledges, accompanied by declamation from high poetry as much as and folk culture, suggests that drinking exists in a historical continuum straddling the adoption of stringent prohibitionist policies; and that ‘being drunk’ and ‘alcoholism’ may be only apparently universal expressions with a different ontohistorical value from what scientific or public policy inquiries or definition suggests.

Consider the question of stigma and moral righteousness. Embracing one’s immorality and ‘bad name,’ as in the case of one’s public intoxication and ‘addiction,’ has been a long-lasting ethical practice in the Iranian context. This is rooted both in the mystical tradition of so-called antinomian movements in Sufism (e.g. *Qalandariyyah* and the *Malamati*, which were active since the 12th centuries and whose cultural influence far outweighed their active membership)^1^; and the socio-literary figure of the *rend*, ‘the libertine cast out’ which is invoked by poets as the highest figure of ethics, the closest to the divine (Ahmed, 2018; Ghiabi, 2022b). In a famous quatrain, the poet Hafez, whose magnum opus is read across the Islamicate world, writes:

‘If, like me, you fall in this trap,

it is enough for you to get hooked^2^ on wine and its cup.

We are lovers, libertine [*rend*], intoxicated [*mast*, also ‘*drunk*’], and world-burning,

Do not sit with us otherwise you will earn a bad name.

There are innumerable examples of this type. What matters in the cultural turn is not the literary or even socio-historical value of the ethics emerging out of this poetical claim. Instead, it is the use of this language and moral code of interpretation to situate experiential and ethical trajectories of ‘addiction,’ into a different ethical order and form-of-life. Invocation of this form-of-life and ethics is a recurrent element in the experience of ‘addiction’ and ‘recovery,’ as we have gathered in our nationwide project. It is also a burgeoning feature in the public engagement of influential ‘healers’ that operate recovery centres and online portals to advise on people’s ‘addiction recovery,’ as studied by Maziyar Ghiabi.^3^

In line with the cultural turn in understanding ‘addiction,’ there is a parallel between alcohol with opium, rather than a dissonance between them, for which we cannot see ‘alcoholism’ as occupying a smaller portion of alcohol use than ‘addiction’ does for opioid use. Alcohol has an equally indigenous cultural trajectory even though distinct, which in turn means that ‘alcoholism’ – i.e. addiction to alcohol – needs to be reclaimed and decentred through cultural situatedness that enables understanding the frame of ‘alcoholism’ as part of a plurality of historical and ethical scripts. The cultural turn in understanding ‘addiction’ could be equally valid for addiction to pharmaceuticals – e.g. ‘pills’ (Behrouzan, 2020) and other such fields such as cannabis (Ghiabi et al., 2018) and ‘gambling addiction,’ though further inquiry are clearly required. What comes to matter, in fact, is that with ‘addiction’ having become a paradigm applied to everything (including coffee, chocolate, Netflix) (Sussman, Lisha & Griffiths, 2011), we risk situating it only as matter of consumption, the existential driver under capitalist forms-of-life (Ghiabi, 2022a). In such conditions, a cultural turn in reclaiming ‘addiction,’ or moving beyond it, is key to appreciate difference and potential for transformative policies.

There have been some instances in which the cultural turn on addiction helped shaping addiction policies in Iran, though only limitedly in a radical way. The most significant of these was the pre-revolutionary opium maintenance policy put in place during the late monarchy of Mohammad Reza Shah (1941–1979) between 1969 and 1979. Here, the policy responded to the cultural place of ‘opium’ and its habit-forming nature through a national plan of opium distribution through physicians and pharmacies, supported by a state monopoly of poppy cultivation (Ghiabi, 2021). A more recent, though less radical intervention was represented by the adoption of extensive harm reduction policies in response to the HIV/AIDS epidemic outbreak, which started in the prisons in the late 1990s and spread out across the country by early 2000s. Harm reduction was clothed in a cultural code in order to be legitimised. This happened by using specific ethical norms in Islamic Shi’a jurisprudence, under the label of *zarurat* (in Farsi; in Arabic *zarurah*), ‘necessity.’ Invoking this jurisprudential notion meant that the Islamic Republic as a form of government driven by religious ethics could take policy decisions that were apparently at odds with the hitherto accepted religious norms. For instance, it meant that public institutions could facilitate and actively engage in needle distribution programmes for heroin users and set up public and private clinics of methadone and buprenorphine maintenance programmes under the governance paradigm of ‘necessity,’ because not doing so would mean a worse health outcome. This occurred also in prisons. ‘In the absence of water in the desert, a Muslim is obliged to drink wine if wine is the only available drink’ was one of the examples that proponents of harm reduction used in their bid to convince reluctant jurists and policymakers (Ghiabi, 2019). However, we should not confuse this culturally coded policy making with the radical cultural turn which we are suggesting, which requires a more profonde, anthropologically informed, and historically situated work to unthink ‘addiction’ as we know it and reclaim it – if necessary – through other onto-epistemic categories.

‘Addiction’ in Iran is today interpreted through the lens of modern biomedical and psychiatric diagnostics. The volume of research produced in addiction sciences by Iran-based researchers is impressive. Their research vision is rooted in the adoption of psychiatric, genetic, molecular, pharmacological techniques and methodologies. Opposed to this, there is a stark dearth of qualitative, culturally informed research on addiction, with questions of historical context, sociological grounds, and the cultural, political environment largely absent. This is surprising given that in many other spheres of the social, political, and cultural life, Iran as a polity has pioneered an indigenous model of evolution, enshrined in the slogan ‘Neither West nor East,’ meaning neither liberal capitalist as in the American sphere, nor Communist as in the Soviet and Chinese spheres. This distinctively antinomian history built upon the pursuit of autocratic development and formulation of self-proclaimed autochthonous codes of moral conduct, as in the case of gender and foreign policies, never materialised into a culturally informed model on health, and specifically ‘addiction.’

For over more than four decades, the Islamic Republic’s language and policy on drugs has not moved away from the orthodox rhetoric and guidelines of the international prohibitionist model, though the country has implemented nation-wide harm reduction policies since the 2000s (Ghiabi, 2019). None of these interventions, either as public policy or as cultural leverage, has delivered generative results and 2020s Iran faces far more serious challenges in dealing with ‘addiction’ than in any previous historical moment. More than four decades of stringent prohibition, which has contemplated life prison sentences for recidivism in drug offences as well as, until recently, the death sentence for drug trafficking, has not impaired the culture of consumption in any tangible way. People use drugs ordinarily and in violation of the law and, based in our nation-wide study (Maarefvand & Ghiabi, 2023), they do not believe that such a behaviour needs to be changed. Why, in light of the above, is prohibition such a stubborn policy even in a political and cultural context that could both ideologically dismiss US-led models and tap into a rich history of experimentation of alternative models of governance of health and life?

Indeed, the place of culture has been the great absent in the epistemic history of ‘addiction.’ This is despite a great deal of formidable anthropological works being written on ‘drugs’ over the past two decades. Rightly so, as this scholarship had emerged in the Americas, the focus had been on racial and socio-economic inequality in societies brunt of their being settler colonies with the annexed epistemic erasure – or in the decolonial jargon *epistemicide* – of other forms of understanding and experiencing ‘addiction.’ Yet, even there one can see the illuminating potential of revisiting the place of culture in the question of ‘addiction,’ for instance in the Latinx and indigenous communities (Garcia, 2008, 2015; Schwartz-Marin & Fiske, 2022). In light of this, we call for a cultural turn in engaging with ‘addiction;’ not because we are dismissing its biomedical aspects, its multi-layered environment of socio-economic and health determinants (Rhodes et al., 2005; Rhodes, 2002; Page & Singer, 2010), or its individual trajectories. This cultural turn is not ‘limited’ to the non-Western world – i.e. the global Majority or global South, though given the near-hegemonic force of West-centric scientific lens of diagnostics, the global South may well be better place to extend a radical alternative to the current regime of knowledge/-production. The cultural turn is an invitation to see whether ‘addiction’ as a modern cultural script can be rewritten through otherwise epistemic and experiential trajectories, including in the colonial and capitalist West where there may be unorthodox forms-of-life that reclaim ‘addiction’ beyond the moral/scientific simplicity of psycho-pharmacological formulas. It is for us as researchers to be open to look, see, read, and compose these alternative modes of living the experience and experiment of ‘addiction,’ when necessary, through a new vocabulary as well as methodological tools. In turn, this can lead the work of policy towards spaces of existence and legitimisation as radically distinctive from the ones more or less universally available now.

## Footnotes

1 It is noteworthy to add that these groups – and the ethics referred to in the figure of the *rend* – are a key cultural component in Hindustani popular culture, as evidenced in the tradition of the *qawwali* in South Asia (Pakistan, Afghanistan, India, and Bangladesh). *Qawwali* is a devotional and ritual form of music originating in South Asian Sufi orders in the 13^th^ century.

2 The word used is *kharab*, which also means ‘ruined,’ hinting at the morally and physically adverse effect of drunkenness.

3 Some of the findings were presented at Maziyar Ghiabi’s Keynote lecture ‘Recovery beyond its possibility of being’ at the Contemporary Drugs Problems conference in Paris, September 2023.
